# A novel ViewRNA in situ hybridization method for the detection of the dynamic distribution of Classical Swine Fever Virus RNA in PK15 cells

**DOI:** 10.1186/s12985-017-0734-4

**Published:** 2017-04-18

**Authors:** Qianyi Zhang, Lu Xu, Yujie Zhang, Tuanjie Wang, Xingqi Zou, Yuanyuan Zhu, Yan Zhao, Cui Li, Kai Chen, Yongfang Sun, Junxiang Sun, Qizu Zhao, Qin Wang

**Affiliations:** National Classical Swine Fever Reference Laboratory, China Institute of Veterinary Drug Control, Beijing, China

## Abstract

**Background:**

Classical swine fever (CSF) is a highly contagious fatal infectious disease caused by classical swine fever virus (CSFV). A better understanding of CSFV replication is important for the study of pathogenic mechanism of CSF. With the development of novel RNA in situ Hybridization method, quantitatively localization and visualization of the virus RNA molecular in cultured cell or tissue section becomes very important tool to address these pivotal pathogenic questions. In this study, we established ViewRNA ISH method to reveal the dynamic distribution of CSFV RNA in PK15 cells.

**Methods:**

We designed several specific probes of CSFV RNA and reference gene β-actin for host PK15 cells to monitor the relative location of CSFV RNA and house-keeping gene in the infected cells. After determining the titer of reference strain CSFV (HeBHH1/95) with the 50% tissue culture infective dose (TCID50), we optimized the protease K concentration and formalin fixation time to analyze the hybridization efficiency, fluorescence intensity and repeatability. In order to measure the sensitivity of this assay, we compared it with the fluorescent antibody test (FAT) and immunohistochemical(IHC) method. Specificity of the ViewRNA ISH was tested by detecting several sub genotypes of CSFV (sub genotype 1.1, 2.1, 2.2 and 2.3) which are present in China and other normal pig infectious virus (bovine viral diarrhea virus (BVDV), porcine parvovirus (PPV), porcine pseudorabies virus (PRV) and porcine circovirusII(PCV-2).

**Results:**

The lowest detection threshold of the ViewRNA ISH method was 10^−8^, while the sensitivity of FAT and IHC were 10^−5^ and 10^−4^, respectively. The ViewRNA ISH was specific for CSFV RNA including 1.1, 2.1, 2.2 and 2.3 subtypes, meanwhile, there was no cross-reaction with negative control and other viruses including BVDV, PPV, PRV and PCV-2. Our results showed that after infection at 0.5 hpi (hours post inoculation, hpi), the CSFV RNA can be detected in nucleus and cytoplasm; during 3–9 hpi, RNA was mainly distributed in nucleus and reached a maximum at 12hpi, then RNA copy number was gradually increased around the cell nucleus during 24–48 hpi and reached the peak at 72hpi.

**Conclusions:**

To our knowledge, this is the first to reveal the dynamic distribution of medium virulence CSFV RNA in PK15 cells using the ViewRNA ISH method. The sensitivity of the ViewRNA ISH was three to four orders of magnitude higher than that of FAT and IHC methods. The specificity experiment showed that the ViewRNA ISH was highly specific for CSFV and no cross-reaction occurred to negative control and other pig infectious virus. This assay is more suitable for studying the CSFV RNA life cycle in cell nucleus. The results proved that CSFV RNA enters into PK15 cells earlier than 0.5hpi, relative to the eclipse period of cytoplasm is 6–9 hpi and CSFV RNA has ever existed in nucleus.

## Background

Classical swine fever (CSF) is a highly contagious fatal infectious disease that primarily affects pigs caused by classical swine fever virus (CSFV) [1–3]. CSF is also one of the notifiable diseases that must be reported by OIE [4]. Due to the great economic losses in pig industry, Chinese government announced priority to prevent and control this major disease and sets the goal of elimination of CSF in all breeder farms by the end of 2020. However, there are still many challenges to achieve this goal [5], for example, currently CSF of China native showed characters of wide prevalence, immunosuppression, high frequency of outbreak and persistent infection. Persistent infection is one of the major reasons that CSF have been long circulating throughout China [6].

Therefore, it is important to study the replication circle which can pay the way to reveal the pathogenic mechanism of CSFV infection [7, 8]. However, the study of virus replication circle, particularly in the cell and viral RNA levels, can constitute an important basis for revealing the infection mechanism. In this study, we established a novel ViewRNA ISH method to reveal the molecular location and dynamic distribution of CSFV on the infected PK15 cells at RNA level.

CSFV attaches the host cell mainly through fusion of its membrane glycoprotein E^rns^ and E2 with the host cell membrane, and then enter the host cell by the receptor-mediated endocytosis [9]. Cell morphology research shows that CSFV replicate mainly in the nucleus periphery surrounding with rich membrane, while mature virions mainly distribute in the amorphous envelope in the cytoplasm. It is well documented that CSFV may release from nucleus periphery and then enter in host cytoplasm [10]. Studies focused on the immune response and viral load in the process of CSFV infection confirmed that CSFV can be infected and replicated in most of pig tissue and organs and viral load is related with the course of disease [11]. However, the dynamic distribution of CSFV RNA is still unknown in the infected cells, or the current methods are ideal enough to study the dynamic distribution of CSFV RNA. For example, electron microscope and IHC can only detect mature virus particle and the detection sensitivity is low. Quantitative real-time PCR can only detect the total RNA, and is not suited to study the dynamics of RNA replication in single infected cell.

ViewRNA ISH is a novel RNA ISH technology developed based on ISH, which has been widely used in cancer study [12, 13], and also is used to verify the existence of new type small RNA virus in Turkey poultry [14]. Recently, researchers studied the replication of Ebola viral RNA in infected cell and found that the virus attach and enter into the target cell less than 6 h, and the RNA completed the replication and release into parietal cell during 12–48dph [15]. In present experiment, we used ViewRNA ISH to study the replication and dynamic distribution of medium virulence strain CSFV RNA in PK15 cell at RNA level, which lays an important theoretical basis for studying CSFV pathogenic mechanism.

## Results

### Optimization of ViewRNA ISH

Considering the fluorescence intensity and repetition are important for the optimum condition of ViewRNA ISH, the best working concentration of protease K and the optimum formalin fixed time were analyzed through orthogonal test. We found the best working concentration of protease K is one thousandth and the optimum formalin fixed time is 30 min. On this condition, it has the best S/N ratio and showed no impact to cellular morphology.

### Sensitivity of ViewRNA ISH

**Fig. 1:**
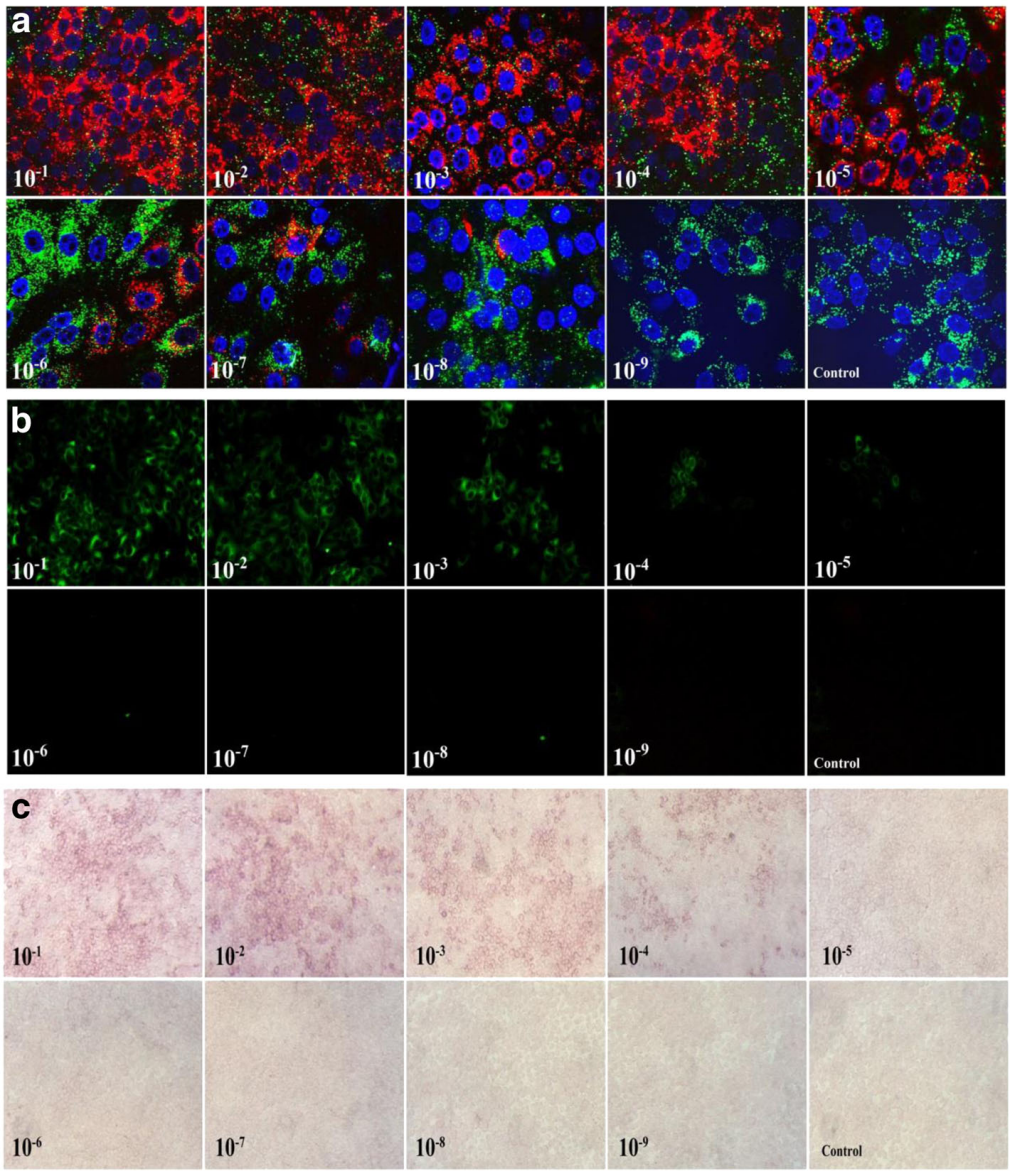
**a** Reaction sensitivity of CSFV ViewRNA ISH. The target gene CSFV RNA dyed with Cy3 show *red bright dot*, internal reference β-actin dyed with FITC show *green bright dot*, nucleus dyed with DAPI show *blue region*. Using negative cell samples without infected as control. **b** Reaction sensitivity of CSFV FAT. Using negative cell samples without infected as control. **c** Reaction sensitivity of CSFV IHC. Using negative cell samples without infected as control

In order to detect the sensitivity of ViewRNA ISH, we compared it with the FAT and IHC method. We used 10-fold serial diluted HeBHH1/95 strain (from 10^−1^ to 10^−10^) that were inoculated in three groups PK15 cells to test the sensitivity of ViewRNA ISH, FAT and IHC method, meanwhile measuring the respective TCID50 by FAT method. The lowest detection threshold of ViewRNA ISH was 10^−8^, also the TCID_50_ of diluted solution is 10^−7.75^/ml, while the sensitivity of the other two methods are 10^−5^ and 10^−4^, and the TCID_50_ of diluted solution are 10^−4.25^/ml and 10^−4.6^/ml, as shown in Fig. 1a, 1b and 1c.

### Specificity of ViewRNA ISH

**Fig. 2:**
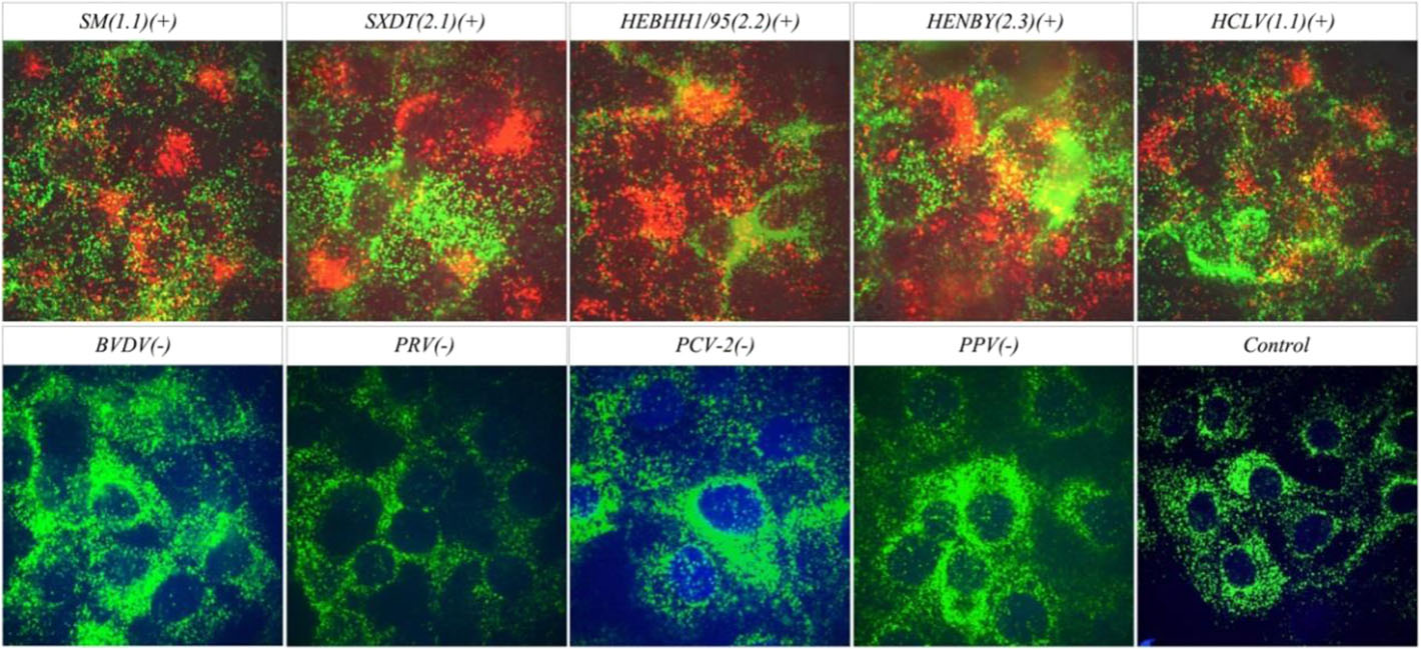
Reaction specificity of CSFV ViewRNA ISH. *Red fluorescent* represents positive siginal of CSFV RNA, *green fluorescent* represents β-actin siginal; Using negative cell samples without infected as control;“+”represcents ISH test result is positive,“-”represcents ISH test result is negative

Other subtypes of CSFV (genotype 1.1, 2.1,2.2 and 2.3) presented in China and other pig infectious virus (BVDV, PPV, PRV, PCV-2) were used to test the specificity by the ViewRNA ISH. Only SM strain(genotype 1.1), C-Strain (genotype 1.1), HeBHH1/95 (genotype 2.1, SXDT2011(genotype 2.2), and HeNBY1/96(genotype 2.3) can be detected by ViewRNA ISH (red fluorescence) in the infected cells. Meanwhile, there is no positive signal in BVDV, PPV, PRV and PCV-2 infected cells (Fig. 2). The results showed that the ViewRNA ISH was highly specific for CSFV and no cross-reaction occurred in negative control and other pig infectious virus.

### Dynamic distribution of HeBHH1/95 detected by ViewRNA ISH

**Fig. 3:**
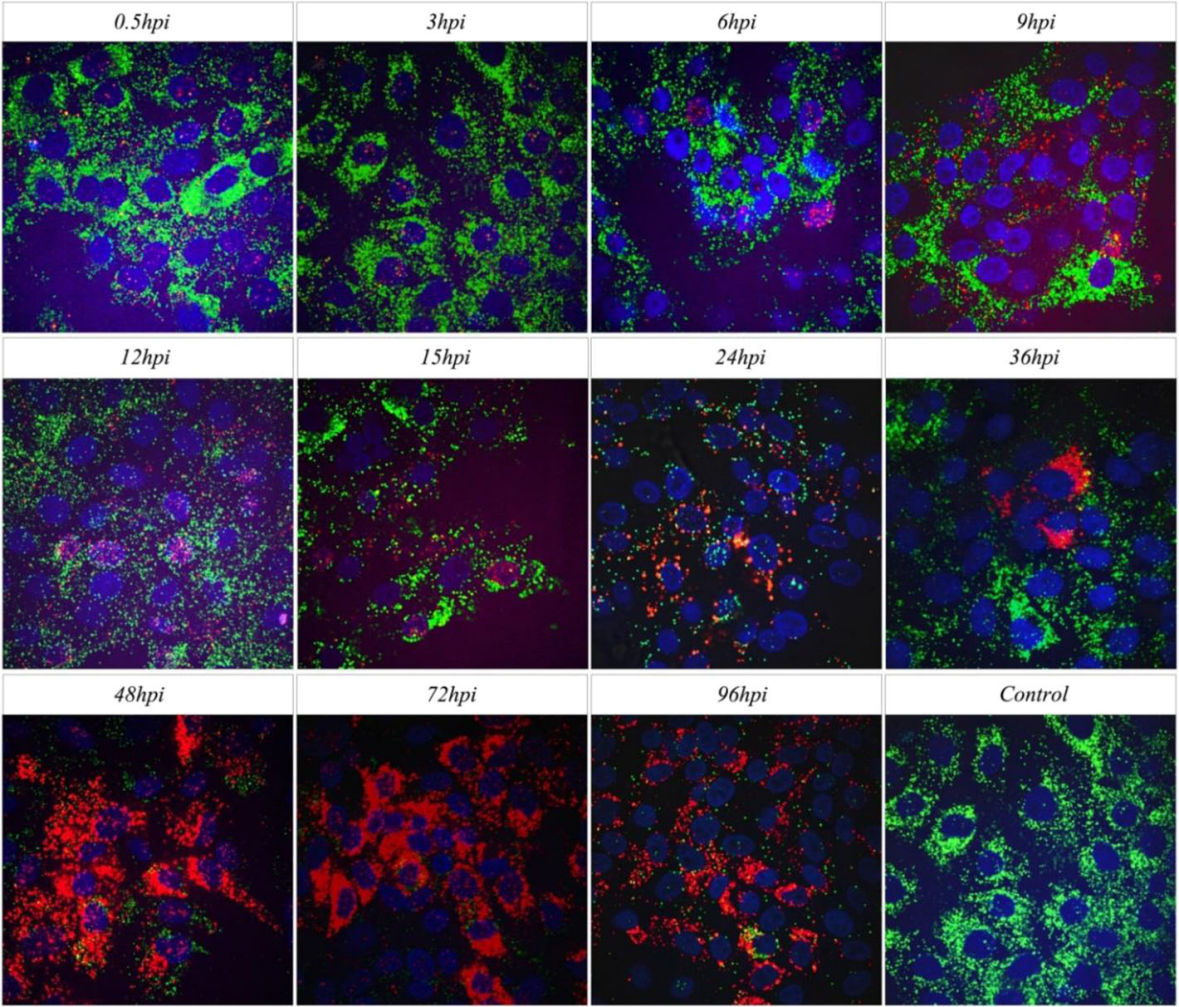
The dynamic distributions of HeBHH1/95 strain RNA in PK15 cells. *Red fluorescent* represents positive siginal of CSFV RNA, *green fluorescent* represents β-actin siginal; Using negative cell samples without infected as control

**Fig. 4:**
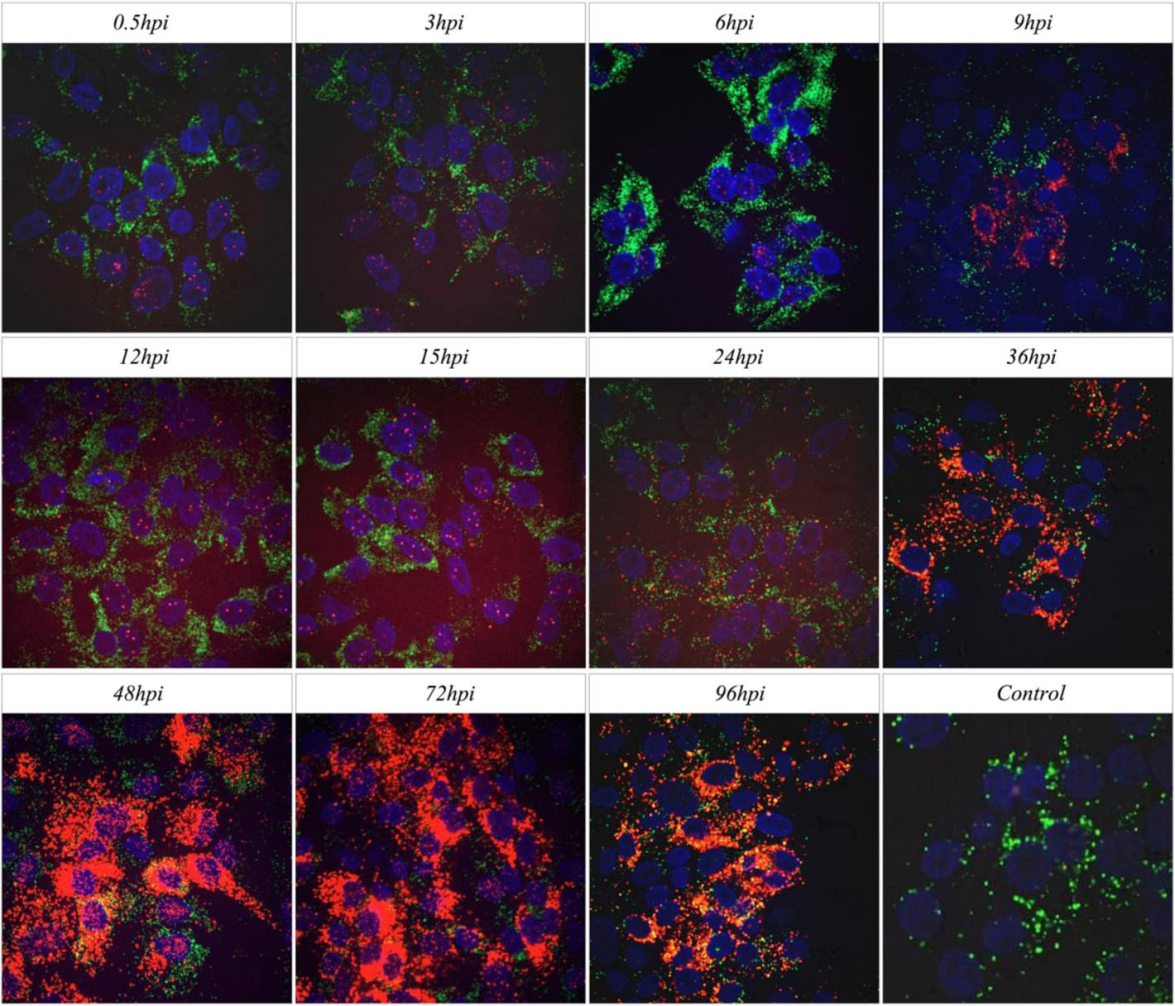
The dynamic distributions of C-strain RNA in ST cells. *Red fluorescent* represents positive siginal of CSFV RNA, *green fluorescent* represents β-actin siginal; Using negative cell samples without infected as control

Different time points of HeBHH1/95 strain infected PK15 cells were used to study the dynamic distribution of CSFV RNA. At early stage of infection, the RNA were detected in the karyon and endochylema after 0.5hpi; during 3 ~ 6hpi, the RNA in the endochylema gradually invades into karyon and the concentrations in the karyon reaches a peak at 12hpi; during 15 ~ 24hpi, the quantity of viral RNA in the endochylema gradually increases and surround nucleus; with lastingness of infection, more and more viral RNA accumulated in the cytolymph during 36–48hpi and with a peak at 72hpi. Until the last 96hpi, the number of viral RNA declined (Fig. 3). Meanwhile, we also used the established ViewRNA ISH method to test the dynamic distribution of C-strain viral RNA in ST cells, it was because the C-strain was defective replication in PK15 cells. The similar results with dynamic distribution of HeBHH1/95 in PK15 cells was obtained (Fig. 4).

### Dynamic distribution of HeBHH1/95 detected by FAT

**Fig. 5:**
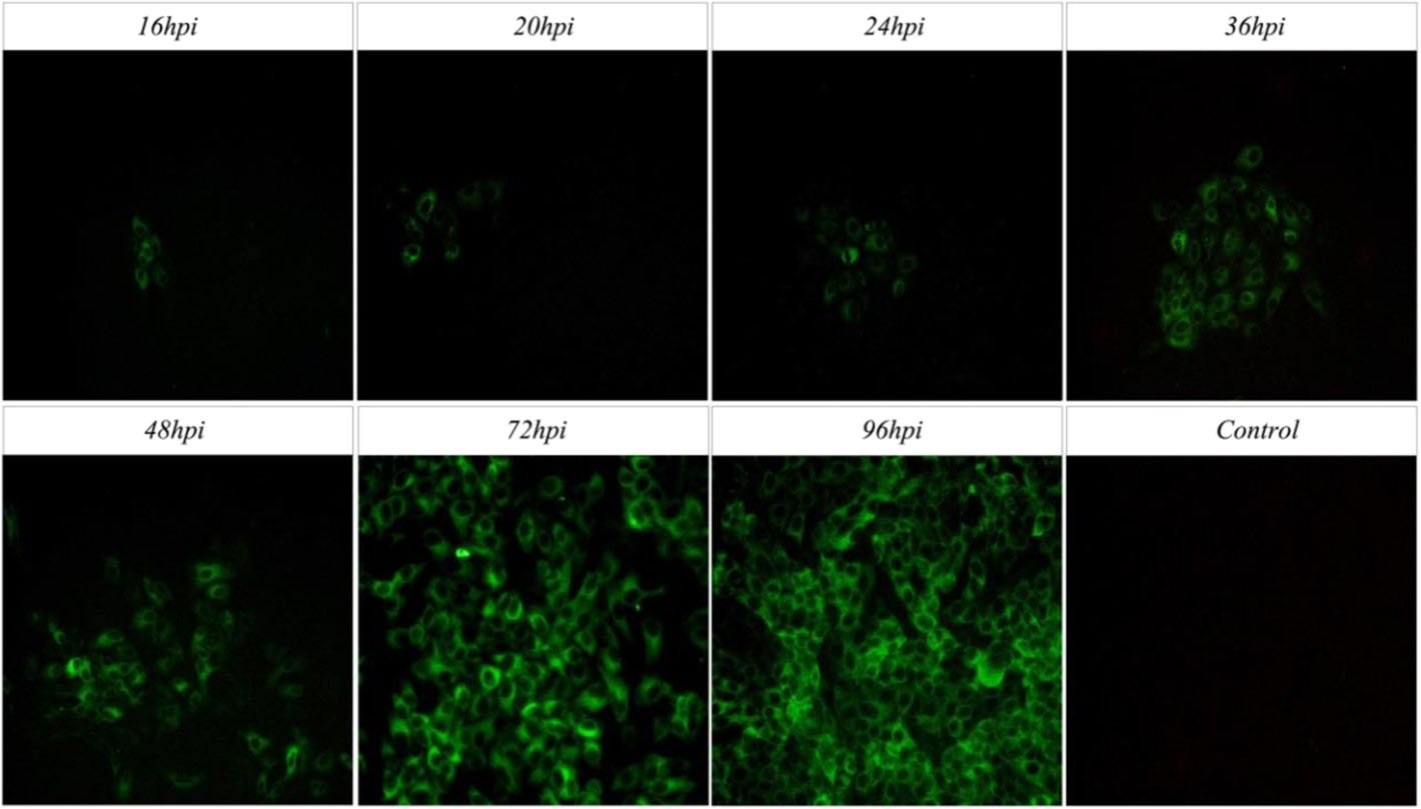
Results of HeBHH1/95 strain E2 protein detected by FAT in PK15 cells. Using negative cell samples without infected as the control

FAT method was also used to study the dynamic distribution of viral E2 protein in the infected PK15 cells to evaluate whether the sensitivity of ViewRNA ISH is better than FAT. Our results showed that the viral E2 protein can be detected with weak fluorescence in minority cell endochylema until 16hpi. With lastingness of infection, the number of positive infected cell constantly increases and with a peak at 72hpi, and then the positive cells declines and the fluorescence degree is weak after 96hpi, as shown in Fig. 5.

## Discussion

In situ hybridization (ISH) is a powerful technique for detecting nucleic acids in cells and tissues. Here we describe ViewRNA ISH that are optimized for detection of CSFV RNA in infected PK15 cell. The operational processes of ViewRNA ISH including The assay includes three steps of sample processing, probe hybridization and visualization.

The relative position of viral RNA and nucleus can be observed under fluorescence confocal microscopy which has the function of obtaining different spectrum section fluorescence signals at the same time and processing obtained cascading effect photos with Volocity Demo software, overcoming the disadvantages of low signal group strength and worse sensitivity of traditional fluorescence in situ hybridization (FISH), and the sensitivity is obviously higher than FAT and IHC. ViewRNA ISH can detect CSFV RNA gradually transferring from karyon to cytoplasm at 6 ~ 9hpi, but FAT was unable to detect the viral E2 protein in cytoplasm until 16 hpi, indicating that ViewRNA ISH is better than FAT and IHC to study the dynamic distribution of CSFV RNA in the infected cell. The specific probe make this method very efficient and there were no cross reactions with BVDV, PPV, PRV and PCV-2 swine virus.

As we know that CSFV entered cells by endocytosis, and replicated in the cytoplasm. The mature viruses were released from infected cells by budding or exocytosis. But viral RNA can also be detected in the karyon at the early infection stage in this experiment, suggesting it may generate some proteins which related with RNA self-duplication in cell nucleus. It was reported that heterogeneous nuclear ribonucleoprotein (hnRNPs) existing in nucleoplasm, taking part in the synthesis processing of viral RNA. For example, hnRNpAI participate in replication of hepatitis C virus. Knockout of the hnRNpAI, the efficiency of HCV RNA replication would be reduced [16]. Some research on CSFV infected PK-15 cells showed that the expression levels of four types hnRNPs were changed indicated the replication of CSFV may require the hnRNPs to synthesis and processviral RNA [17]. In addition, the viral RNA entering into nucleus is related with the nuclear localization of CSFV after infected the host cell [18, 19]. As both flavivirus and CSFV are classified into the same flaviviridae family, it is reported that the nuclear localization of flavivirus C protein in connection with virus replication. The N terminal of C protein may combine with the viral RNA for a few minutes after synthesis and the C terminal of C protein takes part in the formation of nucleocapsid [20]. The CSFV C protein has co-localization with host cell nucleolin (NCL) in nucleolus. When NCL is restrained, the efficiency of CSFV replication is declined [21]. CSFV by acting on nucleus provides convenience for virus replication or regulation of virus [22]. Recently, it was reported that in order to carry out the transcriptional regulation, the core protein may proceed into the nucleus. This speculation is not only reasonable but also possible, because a putative nuclear localization signal(NLS: lys-lys-lys-gly-lys-val), which is located at the carboxyl terminal half of the protein and conserved in different strains of the CSFV virus, was found in the deduced amino acid sequence [23]. Therefore, we suggested that at the early stage of CSFV infection (0–12 h), viral RNA is very likely to be brought into the karyon by C protein. Further studies are needed to study how CSFV RNA enters into nucleus and the function of nucleus.

In addition, the envelope glycoproteins E2 is a non-essential protein of CSFV replication, which appeared behind the appearance of viral RNA. To study the dynamic distribution of CSFV RNA in infected cell, FAT were also used to test the E2 protein dynamic of HeBHH1/95 strain in PK15 cells, which can only detect weak fluorescence in the endochylema until 16hpi, however, ViewRNA ISH is a confocal ISH, which can detect positive fluorescenceat at 0.5hpi. As shown in Fig. 5, viral RNA accumulates outside the nucleus at 36hpi, meanwhile, the number of viral protein E2 detected by FAT in endochylema also increases during 36 ~ 72hpi; the concentrations of extranuclear viral RNA reaches the peak after 72hpi. While the results of FAT also indicates the number of positive cell reaches the peak and the fluorescent intensity is the strongest. After 96hpi, the concentrations of extranuclear RNA is declined and results of FAT also indicates the number of positive cell declines and the fluorescent intensity is weakened, which means the E2 protein concentrations is a positive correlation with the concentrations of viral RNA.

## Conclusions

RNA ISH has been applied to the studies on viral RNA localization of nuclear matrices, using ISH in combination with immunohistochemical method. However, this is the first report that ViewRNA ISH has been applied to the detection of the dynamic distribution of CSFV medium virulence strain (HeBHH1/95) RNA after infected the PK15 cells, which has exhibited some better features such as high sensitivity and specificity. The sensitivity of ViewRNA ISH was higher three and four order of magnitude than FAT and IHC methods, and ViewRNA ISH were highly specific for the detection of CSFV and no cross-reaction occurred in negative control and other pig infectious virus. ViewRNA ISH method seems to be very useful for the detection of the dynamic distribution of CSFV RNA in the early stage of viral infection, which may contribute to the study of CSFV replication and also will be helpful for designing and developing new vaccine. However, there are some drawbacks with this method, such as the ViewRNA ISH reagent are expensive and unmarketed. The results proved that CSFV RNA enters cell early than 0.5hpi, relative to the eclipse period of cytoplasm is 6–9 hpi and RNA has ever existed in nucleus.

## Methods

### Primer and probes

Several specific CSFV probes (P1 is located at 5’-UTR 112 ~ 210 base sequence and P3 is located at NS5B 11426 ~ 11573 base sequence) and labeled probes (P2: label Cy3 red fluorescence) were designed based on CSFV (AF333000) sequence, and the designed probes should be avoid homology regions of bovine viral diarrhea virus (BVDV) (JQ799141) sequence and Border Disease Virus (KC963426) complete sequence. Specific pig house-keeping gene β-actin (ACTB) (AK237086) probe was designed as internal reference with labeled FITC green fluorescence. The design and synthesis of CSFV RNA ISH is completed by classical swine fever reference laboratory and NovoAT&M Biomaterials. The information of the probes sequences:

- P1: GGACTAGCAAACGGAGGGACTAGCCGTAGTGGCGAGCTCCCTGGGTGGTCTAAGTCCTGAGTACAGGACAGTCGTCAATAGTTCGACGTGAGCAGGAGC
- P2: TATGATTTATTGCAAGCCCAGAGGTACGGTATAGAAGACGGGATAAATATCACCAAATCCT
- P3: AGGTGGTCAGACAACACTTCTAGTTACATGCCGGGGAGAAATACAACCACAATCCTAGCTAAAATGGCCACAAGGTTAGATTCCAGTGGTGAGAGGGGTACCATAGCATATGAGAAAGCAGTAGCATTCAGCTTCCTGCTGATGTACT
- ACTB: TTCCTTCCTGGGTATGGAATCCTGTGGCATCCACGAAACTACCTTCAACTCAATCATGAA

### Samples

CSFV shimen strain (SM,F114,1998.6.28), hog cholera lapinizedvaccine (C-Strain), SX-DT/2001 strain, HeBHH1/95 strain and HeNBY1/96 strain are preserved at the China Institute of Veterinary Drugs Control; BVDV (OregonC24V strain, NADL strain), porcine pseudorabies virus (PRV AV1211) and porcine parvovirus (PPV09/79 strain) was kindly provided by China Veterinary Culture Collection Center. Porcinecirco virus II (PCV-2) was kindly provided by Professor Zhou Jiyong of Zhejiang University. The information of these sample is listed in Table 1.

**Table 1:** The samples background information of CSFV RNA ViewRNA ISH

Isolates and vaccines	Genotype	TCID_50_	Infected cell	Source
SM	1.1	10^−3.67^/200 μL	PK15	NCSFRL
C−Strain	1.1	10^−3.5^/200 μL	ST	NCSFRL
SXDT2011	2.1	10^−3.3^/200 μL	PK15	NCSFRL
HeBHH1/95	2.2	10^−4.5^/200 μL	PK15	NCSFRL
HeNBY1/96	2.3		PK15	NCSFRL
PPV09/79			ST	CVCC
BVDV			MBDK	CVCC
PRV			PK15	CVCC
PCV-2			PK15	ZJU

*SM* shimen strain, F1114, *C-Strain* hog cholera lapinizedvaccine, *PPV* porcine parvovirus strain, *BVDV* OregonC24V strain, NADL strain, *PRV* porcine pseudorabies virus, AV1211, *PCV-2* Porcinecirco virus II, *PK15* Pig Kidney Passage Cell Lines, *ST* Swine Testicular Passage Cell Lines, *MBDK* Bovine Kidney Cell; *NCSFRL* National Classical Swine Fever Reference Lab, *CVCC* China Veterinary Culture Collection Center; *ZJU* Zhejiang University

### Reagents

ViewRNA ISH reagent (protease, washing liquor, DAPI) is purchased from NovoAT & MBiomaterials Co, Ltd. The classical swine fever monoclonal antibody (WH303) was kindly provided by England AHVLA Trevor Drew. Lab-Tek II Chamber Slide is purchased from America Thermo Fisher Scientific Company.

### TCID50 of HeBHH1/95 strain

The diluted HeBHH1/95 (from 10^−1^ to 10^−6^) were inoculated in PK15 cell in the 24 well plate, cultured in RPMI 1640 medium with 8% fetal bovine serum (FBS) under 37 °C in 5%CO_2_. After 72 h inoculation, the FAT method was used to measure the TCID_50_ titer.

### Development of ViewRNA ISH

Each well of PK15 cell with 70% ~ 80% densities is inoculated with 200 μL HeBHH1/95 strain with 10^−4.5^ TCID_50_/ml for 72 h in 37 °C under 5%CO2. Addition of 4% formaldehyde to fix under room temperature; incubation for 5 min with washing buffer Solution I after washing with PBS; after addition protease to treat for 15 min and incubation with probe under 40 °C, then add in working concentration DAPI to dye cell nucleus before incubating for 30 min with fluorescence label probe. Finally, controlling the working temperature within 5–20 °C and the wave length is 488/550 nm, using 20 × objective lens for imaging with fluorescence confocal microscopy in the normal temperature evades under light.

### Optimization of ViewRNA ISH conditions

Orthogonal experiment was used to analyze the optimum concentrations of protease K and the most suitable of formaldehyde fixed time. The concentrations of protease K were diluted as: 1:500, 1:1000, 1:2000, 1:4000 and the formaldehyde fixed time is set as: 30 min and 60 min. Considering the test result and fluorescence intensity and repeatability, optimal reaction conditions were determined in Table 2.

**Table 2:** The optimization of proteinase K concentration and the formalin fixed time

Proteinase K concentration	Formalin fixed 30 min	Formalin fixed 60 min
1:500	(-) Probe and (+) Probe	(-) Probe and (+) Probe
1:1000	(-) Probe and (+) Probe	(-) Probe and (+) Probe
1:2000	(-) Probe and (+) Probe	(-) Probe and (+) Probe
1:4000	(-) Probe and (+) Probe	(-) Probe and (+) Probe

“(+)”Represents experimental group with probes,“(-)”Represents negative control without probes

### Sensitivity and specificity

200ul serial dilutions (from 10^−1^ to 10^−10^) of the strain HeBHH1/95 strain (10^−4.5^ TCID_50_) were inoculated in three groups PK15 cells. After inoculation for 72 h at 37 °C under 5%CO_2_, each group was used to detect the viral RNA by ViewRNA ISH,FAT and IHC, respectively, to test their sensitivity.

Different kinds of CSFV, BVDV, PPV, PRV, PCV-2 and other related viral strain quality control sample were inoculated in two groups PK 15 cells. After inoculation for 72 h at 37 °C under 5% CO_2_, two group inoculated cells was used to detect the specificity using ViewRNA ISH and FAT methods.

## Abbreviations

BVDV: Bovine viral diarrhea virus
CVCC: China Veterinary Culture Collection Center
FAT: Fluorescent antibody test
FISH: Fluorescence in situ hybridization
hnRNPs: Heterogeneous nuclear ribonucleoprotein
hpi: Hours post infection
IHC: Immunohistochemical
ISH: In situ hybridization
MBDK: Bovine kidney cell
NCL: Host cellnucleolin
NCSFRL: National Classical Swine Fever Reference Lab
PCV-2: Porcine circovirusII
PK15: Pig kidney passage cell lines
PPV: Porcine parvovirus
PRV: Porcine pseudorabies virus
ST: Swine testicular passage cell lines
TCID50: 50% tissue culture infective dose
ZJU: Zhejiang University

## References

[1] Dahle J, Liess B. A review on classical swine fever infections inpigs: epizootiology, clinical disease and pathology. Comp ImmunolMicrobiol Infect Dis. 1992;15:203–11. 10.1016/0147-9571(92)90093-7 1516362

[2] Taylor DJ, Taylor DJ (1995). Classical swine fever (hog cholera). Pig diseases.

[3] Meuwissen MPM, Horst SH, Huirne RBM, Dijkhuizen AA. A model to estimate the financial consequences of classical swine fever outbreaks: principles and outcomes. Prev Vet Med. 1999;42:249–70. 10.1016/s0167-5877(99)00079-3 10619159

[4] Anonymous. EU Council directive 80/217/EEC on community measures for the control of classical swine fever. 1980.

[5] Terpstra C, de Smit AJ (2000). The 1997–1998 epizootic of swine fever in The Netherlands: control strategies under anon-vaccination regimen. Vet Microbiol.

[6] Stegeman A, Elbers ARW, Smak J, de Jong MCM (1999). Quantification of the transmission of classical swine fever virus between herds during the 1997–1998 epidemic in The Netherlands. Prev Vet Med.

[7] Edwards S, Fukusho A, Lefevre P-C, Lipowski A, Pejsak Z, Roehe P, Wwstergaard J. Classical swine fever: the global situation. Vet Microbiol. 2000;73:103–19. 10.1016/s0378-1135(00)00138-3 10785321

[8] Moennig V (2000). Introduction to classical swine fever: virus, disease and control policy. Vet Microbiol.

[9] Flores EF, Kreutz LC, Donis RO (1996). Swine and ruminant pestiviruses require the same cellular factor to enter bovine cells. J Gen Viro.

[10] Guo L, Wang JS, Chen Z, Yu CC, Tao MH, Li ZJ, Liu Y, Ou WY (2014). CSFV infection characteristics and the analysis of antibody. J Pig Industry.

[11] Liu J, Fan XZ, Wang Q, Xu L, Zhao QZ, Huang W, Zhou YC, Tang B, Chen L, Zou XQ, Sha S, Zhu YY (2011). Dynamic distribution and tissue tropism of classical swine fever virus in experimentally infected pigs. J Virol.

[12] Babij C, Zhang Y, Kurzeja RJ, Munzli A, Shehabedin A, Fenando M, Quon K, Kassner PD, Ruefli-brasse AA, Watson VJ, Fajardo F, Jackson A, Zondlo J, Sun Y, Ellison AR, Plewa CA, Miguel TS, Robinson J, Mccarter J, Schwandner R, Judd T, Carnahan J, Dussault I (2011). STK33 kinase activity is nonessential in KRAS-dependent cancer cells. Cancer Res.

[13] Chae BJ, Bae JS, Yim HW, Lee A, Song BJ, Jeon HM, Chun MH, Jung SS (2011). Measurement of ER and PR status in breast cancer using the QuantiGene2.0 assay. Pathology.

[14] Honkavuori KS, Shivaprasad HL, Briese T, Street C, Hirschberg DL, Hutchison SK, Lipkin WL (2011). Novel picornavirus in Turkey poults with hepatitis, California, USA. Emerg Infect Dis.

[15] Nanbo A, Watanabe S, Halfmann P, Kawaoka Y (2013). The spatio-temporal distribution dynamics of Ebola virus proteins and RNA in infected cells. Sci Rep.

[16] Kim CS, Seol SK, Song OK, Park JH, Jang SK (2007). An RNA—binding protein, hnRNP A1, and a scaffold protein, sptin 6, facilitate hepatitis C virus replication. J Virol.

[17] Sun JF, Shi ZX, Guo HC, Tu CC (2008). Swine fever virus infection induced PK 15 cells proteome changes. Chinese Association of Animal Science and Veterinary Medicine(CAAV).

[18] Mori Y, Okabayashi T, Yamashita T, Zhao Z, Wakita T, Yasui K, Hasebe F, Tadano M, Konishi E, Matsuura Y (2005). Nuclear localization of Japanese encephalitis virus core protein enhances viral replication. J Virol.

[19] Tsuda Y, Mori Y, Abe T, Yamashita T, Okamoto T, Ichimura T, Moriishi K, Matsuura Y (2006). Nucleolar protein B23 interacts with Japanese encephalitis virus core protein and participates in viral replication. Microbiol Immunol.

[20] Tu YX (2005). Research of CSFV E2 genic clone and immune effect of DNA vaccine.

[21] Zhang X, Shi HY, Xu J (2015). Characterization of interaction between the capsid protein of classical swine fever virus and cellular nucleolin. Chin J Prev Vet Med.

[22] Liu N, Shi HY, Chen JF, Feng L, Zhang X, Hu GX (2014). Nucleus localization of classical swine fever virus capsid proteins in cells. Chin J Prev Vet Med.

[23] Liu JJ, Wong ML, Chang TJ (1998). The recombinant nucleocapsid protein of classical swine fever virus can act as a transcriptional regulator. Virus Res.

